# Prognostic significance of ^18^FDG PET/CT in colorectal cancer patients with liver metastases: a meta-analysis

**DOI:** 10.1186/s40644-015-0055-z

**Published:** 2015-11-20

**Authors:** Qian Xia, Jianjun Liu, Cheng Wu, Shaoli Song, Linjun Tong, Gang Huang, Yuanbo Feng, Yansheng Jiang, Yewei Liu, Ting Yin, Yicheng Ni

**Affiliations:** Department of Nuclear Medicine, Ren Ji Hospital, School of Medicine, Shanghai Jiao Tong University, No.160, Pu Jian road, Shanghai, 200127 China; Department of Health Statistics, Second Military Medical University, No. 800, Xiang Yin road, Shanghai, 200433 China; Department of Imaging and Pathology, University Hospitals, KU Leuven, Herestraat 49, Leuven, 3000 Belgium

**Keywords:** SUV, Meta-analysis, Liver metastases, PET /CT, Prognosis

## Abstract

**Background:**

The role of 18-fluorodeoxyglucose positron emission tomography CT (^18^FDG PET/CT), as a prognostic factor for survival in colorectal cancer patients with liver metastases, is still controversial. We sought to perform a meta-analysis of the literature to address this issue.

**Methods:**

A systematic literature search was performed to identify the studies that associated ^18^FDG PET/CT to clinical survival outcomes of patients with liver metastases. Methodological qualities of the included studies were also assessed. The summarized hazard ratio (HR) was estimated by using fixed- or random-effect model according to heterogeneity between trails.

**Results:**

By analyzing a total of 867 patients from 15 studies, we found that PET/CT for metabolic response to the therapy was capable of predicting event-free survival (EFS) and overall survival (OS) with statistical significance, and the HR was 0.45 (95 % confidence interval [CI], 0.26–0.78) and 0.36 (95 % CI, 0.18–0.71), respectively. Furthermore, pre-treatment ^18^FDG PET/CT with high standardized uptake value (SUV) was also significantly associated with poorer OS HR, 1.24; (95 % CI, 1.06–1.45). However, we did not find a statistically significant effect of post-treatment SUV for predicting OS HR, 1.68; (95 % CI, 0.63–4.52).

**Conclusions:**

The present meta-analysis confirms that ^18^FDG PET/CT is a useful tool to help predict survival outcomes in patients with liver metastases.

**Electronic supplementary material:**

The online version of this article (doi:10.1186/s40644-015-0055-z) contains supplementary material, which is available to authorized users.

## Background

The liver is the most common site for hematogenous spreading of metastatic neoplasms. Metastases can result from a wide variety of malignancies, with the most widely known being from colorectal origins. Previous study indicated that liver metastases were detected in 40–50 % of nearly one million patients who were diagnosed with colorectal cancer worldwide each year [1]. In the past, only 10 % of such patients with liver metastases were eligible to surgery. New chemotherapy regimens and improvement in surgical techniques have now allowed surgically treating patients with liver metastases in more advanced stages of illness. Radioembolization using yttrium-90 (^90^Y) resin, also known as selective internal radiation therapy (SIRT), is a palliative treatment, which reduces the liver tumor mass and might eventually permit surgical resection. However, treatment outcomes of liver metastases remain heterogeneous. Therefore, finding reliable prognostic indicators or biomarkers, especially with those non-invasive imaging methods, would be very helpful in the management of patients with liver metastases, which has aroused great research and clinical interests.

Over the recent decades, 18-fluorodeoxyglucose positron emission tomography (^18^FDG PET/CT) has played an increasing role in clinical management of liver metastases. Unlike traditional anatomical imaging modalities, PET/CT can provide not only morphological but also functional information in a single session [2]. In particular, ^18^FDG as a glucose analogue has become the most popular PET tracer to visualize abnormal glucose metabolism in oncology. Because enhanced glucose metabolism is related to the aggressiveness of cancer cells, ^18^FDG PET/CT yielded superior results in monitoring therapy response and predicting survival in patients with a range of malignant tumors [3–7]. The changes in tumor size and/or tumor number, which reflect the number of neoplastic cells, can be used as a radiological or anatomic indicator of the tumor response [8]. Nowadays, ^18^F-FDG PET/CT has been used frequently for the assessment of therapeutic responses. It is particularly preferred over anatomical imaging in patients receiving noncytotoxic therapy as it returns to normal much more quickly [9]. Although the tumoral ^18^F-FDG uptake tends to vary for a number of reasons, there is a direct correlation between ^18^F-FDG uptake and viable tumor cells. In addition, previous research [10] found a significant agreement between anatomic and metabolic criteria. Recent studies have correlated high ^18^FDG uptake in tumors with poorer patient survival in lung, breast, head and neck, and esophageal cancers [11–13]. Furthermore, pretreatment tumoral ^18^FDG uptake has been shown to represent an independent prognostic factor in patients with liver metastases undergoing whatever primary treatment modalities [14, 15]. At present, a frequently used quantitative method in PET is the standardized uptake value (SUV). Compared with various other quantitative approaches, clinical appeal of SUV lies in its simplicity and high reproducibility, thanks to implemented modern computer software. It plays an important role in the evaluation of patient responses to therapies. Practitioners rely heavily on changes in SUVs over time, in the absence of clear improvement or progression of disease by detection of previous lesions or new lesions, respectively, to decide whether to continue or switch therapies. Thus, not surprisingly, an overwhelming volume of SUV data has accrued in PET-related oncologic literature during recent decade. However, the results about the prognostic value of SUV from ^18^FDG-PET/CT in colorectal cancer patients with liver metastases remain uncertain due to small sample size, various inclusion criteria, and different data analysis strategies. Therefore, through a current literature review, for the first time to our knowledge, we have performed a meta-analysis to assess the prognostic value of SUV from ^18^FDG-PET/CT for the survival of patients with liver metastases.

## Methods

### Search strategy

We searched PUBMED, EMBASE, and MEDLINE for articles published between January 2000 and March 2015 to identify the studies evaluating the prognostic value of ^18^FDG PET/CT in long-term survival prediction for patients with liver metastases. We used following search terms: liver metastases, ^18^FDG PET/CT, prognosis, and survival. If overlapping patient cohorts were used between several studies, we only retained the latest or the largest study to avoid duplication of information. When key information for meta-analysis was missing, we contacted the researchers of selected studies for supplying additional data. Only articles published in English were included. As this analysis is merely a retrospective literature review, neither Institutional Review Board approval nor informed consent was required by national and regional laws.

### Inclusion criteria

The eligibility of abstracts and full texts were assessed by three reviewers independently. Disagreements were resolved by discussion. Studies on which agreement could not be accessed were all included for full text screening. According to a previous report [16], the relevant studies were manually selected carefully based on the following criteria: 1) include more than 10 colorectal cancer patients with liver metastases who underwent treatments; 2) use once ^18^FDG PET/CT scan (pretreatment or post-treatment) or twice ^18^FDG PET/CT scans (pretreatment and post-treatment) to predict the survival of patients, where PET/CT was evaluated alone or in comparison to other tests; 3) report prognostic outcomes such as overall survival (OS), progression-free survival (PFS), disease-free survival (DFS) and relapse-free survival (RFS), and compare the outcomes between positive and negative results of ^18^FDG PET/CT; and 4) contain extractable survival data of hazard ratio. However, reviews and other editorial materials were excluded and the studies only focusing on the performance of ^18^FDG PET/CT in diagnosis, staging, and monitoring recurrence or metastasis were also excluded. In addition, reports concerning patients with suspected or diagnosed recurrent disease were not adopted.

### Qualitative assessment

Three investigators reviewed all the publications to assess their methodological quality, determine their eligibility for inclusion in the quantitative meta-analysis and extract the most important information determining the clinical and ^18^FDG PET/CT characteristics. As shown in Table 1, a methodological quality scale has been adapted from a previous study [17] for the purpose of this study using the variables available from the publications. The clinical report and ^18^FDG PET/CT report were scored on 30 and 18 points respectively. A value between 0 and 2 was attributed to each item. When items were not applicable to a particular study, they were ruled out. The scores were expressed in percentage of the maximal theoretical value that could be obtained.

**Table 1:** A methodological quality scale of publications

Assessment parameter			Scale	
Clincal reports	Prognostic factors	Age	0	2
		Gender	0	2
		Performance status	0	2
	Tumor characteristics	Location of primary tumor	0	2
		Number of metastatic sites	0	2
		Size of hepatic metastasis	0	2
		Differentiation grade	0	2
	Description of the results of survival analysis	Number of patients	0	2
		Number of deaths	0	2
		Follow-up duration	0	2
		Number of patients lost to follow-up	0	2
		Univariate and multivariate analyses	0	2
		Description of statistical tests	0	2
		Survival definition	0	2
		SUV cutoff definition or response definition	0	2
Subscores				
PET/CT reports	Patients’ Characteristics	Weight	0	2
		Glycaemia	0	2
	^18^FDG PET acquisition protocol characteristics	Injected dose of ^18^FDG	0	2
		Delay between injection	0	2
		Data acquisition	0	2
		Fasting duration	0	2
	Technical Parameters	SUV formula	0	2
		Type of PET/CT engine	0	2
		Type of SUV attenuation and reconstruction parameters	0	2
Subscores				
Total scores				

### Data extraction and analyses

From those selected articles, two independent reviewers extracted the data that include the first author, publication year, study design, sample size, PET/CT timing, type of treatment received and end points for evaluating the prognostic performance. Specifically, PFS or RFS were merged as one outcome newly defined as event-free survival (EFS), which was measured from the date of therapy initiation to the date of recurrence or progression. We took overall survival (OS) as another outcome index. Patients underwent once PET/CT scan (pre- or post-treatment) were divided into high SUV and low SUV groups according to SUV cutoff values or visual observation. In patients underwent twice PET/CT scans (pre-treatment and post-treatment), the difference between baseline and follow-up SUV values (ΔSUV) was used to differentiate responding from nonresponding based on the definition in each individual study. Survival data from all studies were analyzed by means of the Kaplan-Meier curves, unless hazard ratios (HRs) were reported and compared to calculate HR and 95 % confidence intervals (CI) as previously described by Parmar et al. [18] and Tierney et al. [19]. These effects were combined to compare the low SUV or metabolic responding and high SUV or metabolic nonresponding arms for the overall effect. If the numerator and denominator appeared opposite in an article, we chose to use ln(HR) and the generic inverse variance method in software Review Manager 5.3.0 to get a result. HR < 1 indicated the survival benefit from a metabolic responding low SUV, whereas HR > 1 denoted an increased risk of metabolic nonresponding progression and death. It is considered statistically significant when a P-value is less than 0.05. Statistical heterogeneity was measured using the chi-square *Q* test, while P < 0.10 was considered to represent significant statistical heterogeneity, and expressed by the I^2^ statistic, as described by Higgins et al. [20]. Funnel plot was performed for testing publication bias. Survival rates on the graphical survival curves were read by software Engauge Digitizer version 4.1. (Trolltech, Oslo, Norway). HRs and their variations were calculated by Review Manager 5.3.0 (The Nordic Cochrane Centre, Copenhagen, Denmark).

## Results

### Study inclusion and characteristics analyses

**Fig. 1:**
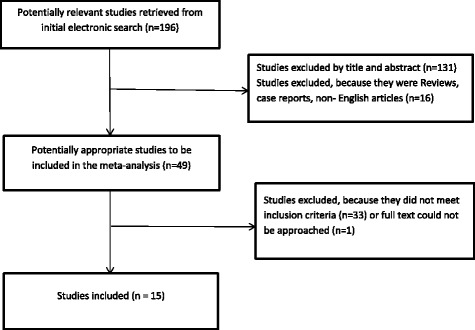
Flow chart of the studies selection process

An internet-based electronic search resulted in 196 potentially eligible articles from all databases. Among them, 147 articles were excluded based on their abstracts, including 3 non-English articles, 4 case reports, 7 reviews, and 133 articles that were irrelevant to prognostic performance of ^18^FDG PET/CT in patients with liver metastases. The remaining 49 full-text articles were further analyzed for eligibility. Of these studies 33 were excluded because the log HR and its variance could not be calculated and one article was only excluded for inaccessible full text. Finally, a total of 15 studies were determined to be qualified for the actual meta-analysis. Figure 1 illustrates the inclusion process and reasons for exclusion. Finally, a total of 867 patients from these 15 studies were analyzed, and the characteristics of selected studies are described in Table 2. The main SUV and ΔSUV characteristics reported in the publication are presented in Tables 3 and 4.

**Table 2:** Principal characteristics of the 15 studies included in the meta-analysis

Study	Publication year	Number of patients	Study design	PET/CT timing	Type of treatment	End points^a^	Methodology score (%)
de Geus-Oei L F et al.	2006	152	Retrospective	Pretreatment	Resection or Chemotherapy	OS	83.33 %
Small RM et al. [21]	2009	54	retrospective	Pretreatment and posttreatment	Chemotherapy	OS EFS(PFS)	50.00 %
Hendlisz A et al.	2011	41	Prospective	Pretreatment and posttreatment	Chemotherapy	OS EFS(PFS)	70.83 %
Muralidharan V et al.	2012	30	retrospective	Pretreatment	Resection	OS EFS(RFS)	75.00 %
De Bruyne S et al. [22]	2012	19	Retrospective	Pretreatment and posttreatment	Chemotherapy and bevacizumab	EFS(PFS)	75.00 %
Lastoria S et al.	2013	33	Retrospective	Pretreatment and posttreatment	Chemotherapy and Bevacizumab	EFS(PFS) OS	66.67 %
Mertens J et al. [23]	2013	18	Prospective	Pretreatment and posttreatment	Chemotherapy and Bevacizumab	OS	75.00 %
Zerizer I et al.	2013	25	Retrospective	Pretreatment and posttreatment	^90^Y radioembolization	EFS(PFS)	70.83 %
Fendler W P et al.	2013	80	Retrospective	Pretreatment and posttreatment	^90^Y radioembolization	OS	62.50 %
Jones C et al.	2014	79	retrospective	Pretreatment	Resection	OS	54.17 %
Lee HS et al. [24]	2014	120	retrospectively	Pretreatment	Resection	OS EFS(RFS)	83.33 %
Lau LF et al.	2014	37	retrospective	Pretreatment and posttreatment	Chemotherapy	OS EFS(RFS)	58.33 %
Riedl CC et al. [25]	2007	90	retrospective	Pretreatment	Resection	OS	54.17 %
Correa-Gallego C et al.	2015	38	Prospective	Pretreatment and posttreatment	Chemotherapy and bevacizumab	OS EFS(PFS)	83.33 %
Sabet A et al.	2015	51	retrospective	Pretreatment and posttreatment	^90^Y radioembolization	OS	70.83 %

^a^"end points": time elapsed between treatment initiation and disease progression or death

**Table 3:** Main SUV (pretreatment or posttreatment) characteristics extracted from the 9 articles used for meta-analysis

study	Type of SUV	Correction of SUV	Threshold definition	SUV threshold
de Geus-Oei L F et al.	preSUVmean	Body weight	Median	>4.26
Muralidharan V et al.	preSUVmean	Body weight	Best cut-off	>10
De Bruyne S et al.	postSUVmax	Body weight	Median	>2.85
Mertens J et al.	postSUVmax	Body weight	Median	2.85
Claire Jones.et al.	PreSUVmean	Body weight	Arbitrary	>7.0
Lee HS et al.	Pre^a^nSUVpeak	Body weight	Median	>5.0
Lau LF et al.	postSUVmax	Body weight	Previous report	>10
Riedl CC et al.	preSUVmax	Body weight	Best cut-off	>10
Correa-Gallego C et al.	preSUVmax	Body weight	Median	>10

^a^nSUVpeak: normalized SUVpeak

**Table 4:** Main ΔSUV characteristics extracted from the 8 articles used for meta-analysis

Study	Type of SUV	Correction of SUV	Threshold definition	SUV threshold
Small RM et al.	^a^ΔSUV	Body weight	Visual observation	unclear
Hendlisz A et al.	ΔSUVmax	Body weight	^b^EORTC	metabolic decrease > 25 %
De Bruyne S et al.	ΔSUVmax	Body weight	EORTC	metabolic decrease > 25 %
Lastoria S et al.	ΔSUVmax	Body weight	Median	metabolic decrease > 50 %
Zerizer I et al.	ΔSUVmax	Body weight	Best cut-off	metabolic decrease > 2.0
Fendler W P et al.	ΔSUVmax	Body weight	^c^PERCIST	metabolic decrease > 30 %
Correa-Gallego C et al.	ΔSUVmax	Body weight	^d^WHO	metabolic decrease > 25 %
Sabet A et al.	ΔSUV	Body weight	Best cut-off	metabolic decrease > 50 %

^a^ΔSUV : difference between baseline and follow-up SUVmax values

^b^EORTC: The criteria of the European Organisation for Research and Treatment of Cancer

^c^PERCIST: PET Response Criteria in Solid Tumors

^d^ WHO: WHO Criteria

### Qualitative assessment

Overall, the publications’ quality score ranged from 50 to 83.33 %, with a median score of 68.33 % (Table 2). If necessary, we attempted to contact the authors to gain missing details of the methodological quality.

### Meta-analyses

#### Predictive value of ΔSUV for EFS

**Fig. 2:**
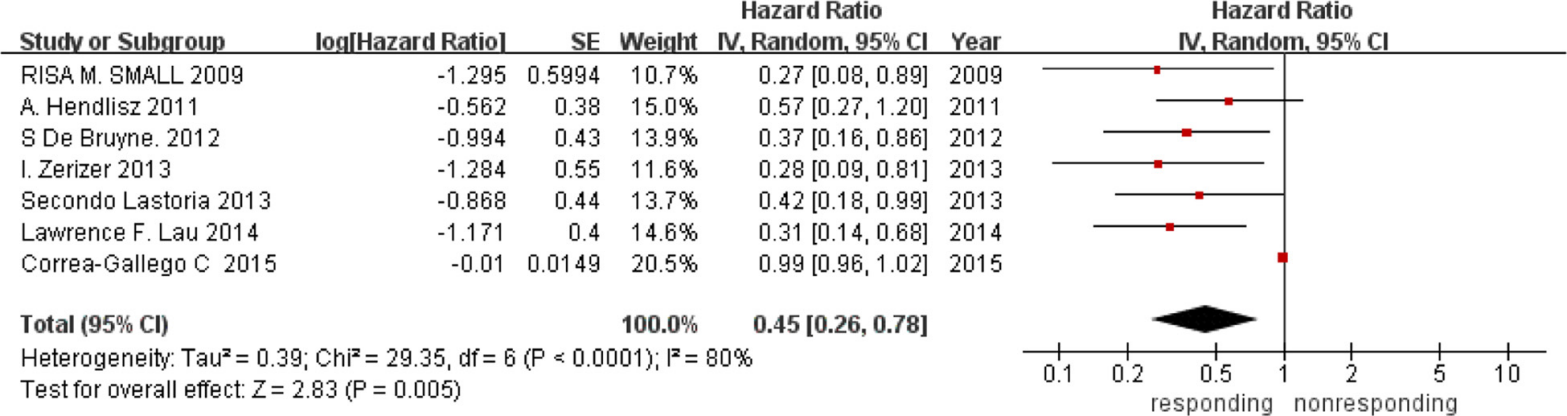
Forest plot of 7 included studies in ΔSUV for EFS. Pooled effect (HR) and heterogeneity test of a metabolic responding on EFS in patients with liver metastases (EFS: event-free survival; HR: hazard ratio)

A total of 7 studies focused on predictive value of ΔSUV for EFS, 5 studies showed that there were significant differences between responding and nonresponding groups for EFS prognosis, 1 study did not indicate this outcome. Figure 2 shows the results of meta-analysis of the 7 studies containing 247 patients comparing EFS in the responding group with that in the non-responding group (P < 0.0001). With a summarized HR less than 1, it suggests a survival advantage for the responding group. We find heterogeneity between the studies (I^2^ = 0 %, P = 0.84). In pooled analysis, EFS was significantly better in responding group, 0.45 (95 % CI, 0.26–0.78) by random model.

#### Predictive value of ΔSUV for OS

**Fig. 3:**
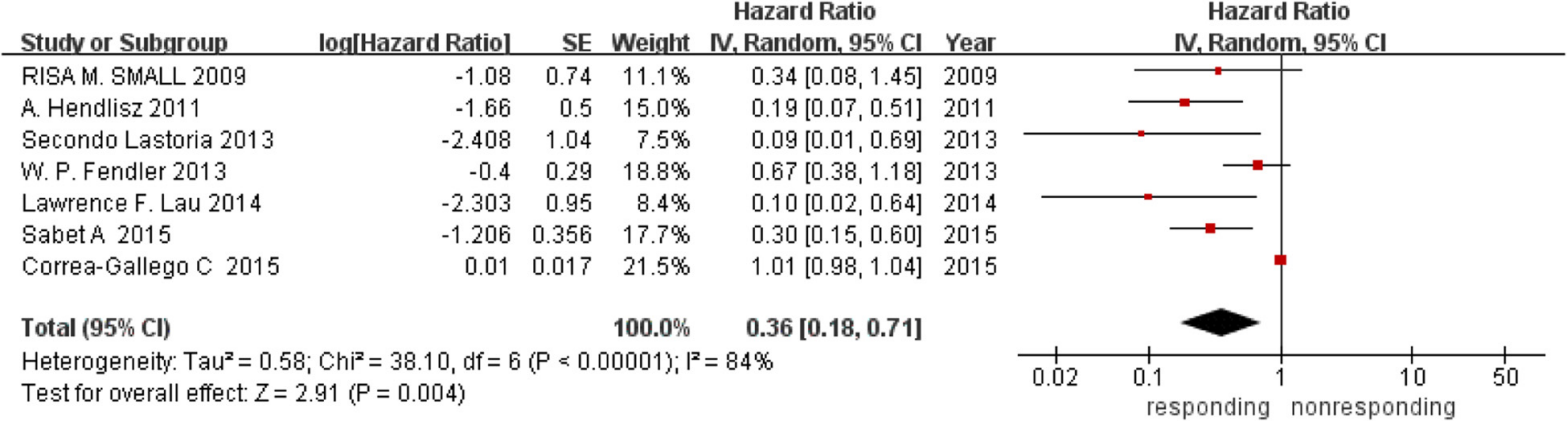
Forest plot of 7 included studies in ΔSUV for OS. Pooled effect (HR) and heterogeneity test of a metabolic responding on OS in patients with liver metastases (OS: overall survival)

Seven studies covering 334 patients were analyzed in this comparison. With I^2^ = 82 %, the heterogeneity could not be ignored between the studies. Thus, we chose random model to calculate the summarized HR, 0.36 (95 % CI, 0.18-0.71), suggesting that the OS was significantly better with responding group (P = 0.004) as revealed in Fig. 3.

#### Predictive value of pretreatment SUV for OS

**Fig. 4:**
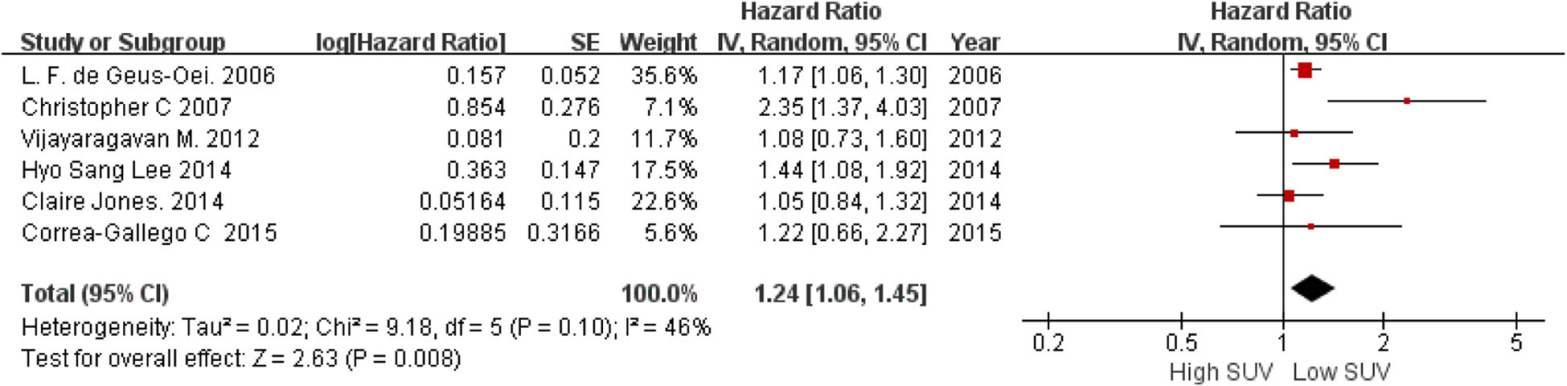
Forest plot of 6 included studies in pretreament SUV for OS. Pooled HR compared low SUV group with high SUV group in patients with liver metastases

As displayed in Fig. 4, six studies involving 509 patients were evaluated in this comparison. The OS disadvantage for high SUV PET/CT images over low SUV ones was statistically significant (P = 0.008), the test for heterogeneity was no statistically significant (I^2^ = 46 %, P = 0.10). By fixed-effects model with a summarized HR, 1.24 (95%CI, 1.06–1.45), it suggests that high SUV PET/CT results were associated with poor OS.

#### Predictive value of post-treatment SUV for OS

**Fig. 5:**
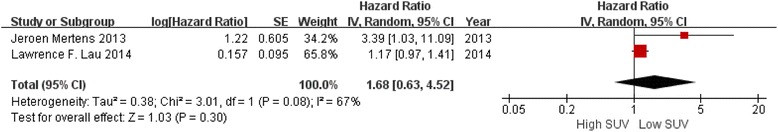
Forest plot of 2 included studies in posttreament SUV for OS. Pooled HR compared low SUV group with high SUV group in patients with liver metastases

Three other studies including 55 patients were assessed to estimate the prognostic value for predicting OS by post-treatment PET/CT. The results are demonstrated in Fig. 5, indicating that there was no statistically significance between high SUV PET/CT group and low SUV PET/CT group for OS, HR, 1.68; (95%CI, 0.63–4.52), P = 0.30. There was significant heterogeneity between researches (I^2^ = 67 %; P = 0.08).

## Discussion

Recently, the degree of tumor uptake of ^18^FDG on PET as assessed by the SUV was shown to be an independent prognostic factor in liver metastases [26, 27], but some studies have not found a significant association between SUV and prognosis [28–30]. Apparently, there have been controversial results. Therefore, we have elaborated this meta-analysis to evaluate the prognostic value of ^18^FDG PET-CT for liver metastases.

This meta-analysis has provided two meaningful findings regarding the use of ^18^FDG-PET for predicting survival of patients with liver metastases. First, some studies do have addressed the predictive value of patients with metabolically nonresponding liver metastases after treatment for OS and EFS. Through our quantitative analysis, the risk of death in nonresponding group was 2.5 times as high as the responding group in OS and 2.632 times in EFS. We assumed that these tumors are more aggressive or invasive with hyperactive metabolism as displayed in ^18^FDG PET/CT images. Although a range of factors has been associated with ^18^FDG uptake, there appears to be a rather strong relationship between ^18^FDG uptake and cancer cell mass in a number of studies [31, 32]. The metabolic response, which might be the strongest marker of prognosis, was measured in tumors that were ultimately resected. It appeared that the value of metabolic response was not just for the evaluation of the visible hepatic lesions but also for serving as an indicator for the sensitivity to chemotherapy among potentially undetectable micro-metastases. Current imaging technologies have limitations in resolution, with PET systems being able to detect tumors as small as 0.3–0.7 cm. This corresponds to a minimum detectable tumor size with about 10^8^–10^9^ cells [33]. Consequently, it is reasonable that declined tumor SUV would be seen with a loss of viable cancer cells, and increased tumor glucose use and volume of tumor cells would be expected in progressive tumors. The evaluation of metabolic response before planned resection of liver metastases includes the strengths of being noninvasive, repeatable, quantifiable and discriminative towards clinical outcomes. At present, ^18^FDG PET/CT appears to be the best prognostic tool available for the oncologists in the field of hepatobiliary surgery [34].

Secondly, our meta-analyses on the prognostic value of pre-treatment SUV measured on metastatic sites also showed that high SUV was an independent prognostic factor for poorer OS. By quantitative analyses, the risk of death in high SUV group was 1.12 times the low SUV group in OS (95%CI 0.01–0.25, P = 0.04), no matter whether patients subsequently underwent curative surgery or chemotherapy. This is most likely due to the fact that high SUV tumors are more aggressive and metastatic, thus leading to relatively worse prognosis. One-unit increase in SUV could correspond to a significant increase of 17 % in the risk of death [35]. Thus, our data suggest that intense glucose metabolism in liver metastases is a negative marker of prognosis. However, there existed large discrepancies among the cutoff values to distinguish high SUV from low SUV PET results (thresholds varying from 2.85 to 20). Higashi et al. [36] and Vansteenkiste et al. [37] indicated that dichotomization with a wide-ranging of SUVs gave significantly discriminative log-rank probability values. This suggests that the relationship between SUV and prognosis could be a gradual one rather than based on a threshold. The wide range of SUV thresholds seen in these articles can be due to several factors such as institution-based technical variations, the heterogeneity of the patient cohorts analyzed and the variance in the PET scanners and acquisition protocols used.

As subjective criteria were used in some studies (Table 3), despite such variability, we were able to show that the SUV was indeed correlated with patient survival. In our study, we summarized an HR from individual articles based on the SUV threshold used in each particular study, which helped to cancel the threshold factor to a certain extent. By doing so, we could demonstrate that the SUV was surely worth considering as a prognostic factor in patients with liver metastases. In addition, only three studies dealing with the predictive value of post-treatment SUV for OS were included. One of these two studies with a total of 18 patients indicated significant OS benefit of low SUV after treatment, but another study with 37 patients showed an opposite conclusion. Summary of results showed that between high SUV and low SUV groups, no statistically significant effect was found (95 %, CI 0.63–4.52; P = 0.08). This lack of significance could be explained by insufficient study inclusion for the present analyses, which did not allow a definite conclusion in this aspect. In future studies, we should collect more similar type of studies for more comprehensive research.

There exist a number of limitations in our study. First, our meta-analysis was restricted to articles published only in English. Secondly, the methodology used did not prevent all of the potential biases (e.g. Additional file 1: Figure S1; Additional file 2: Figure S2; Additional file 3: Figure S3; Additional file 4: Figure S4), which was partially due to the fact that studies with nonstatistically significant results were less often published. Third, the clinical heterogeneity may also account for the test results, since among included studies patients received different therapies (e.g. various chemotherapy schemes with or without bevacizumab and the types of surgery or surgical techniques) that may all influence the outcomes. Last but not least, some of the included studies did not provide sufficient data of time-to-event outcomes for meta-analysis directly. We had to use Engauge Digitizer to extract data from survival curves, which may result in certain inaccuracy. Nevertheless, the validity of the major findings in this study has been supported by more recent clinical studies [38, 39].

## Conclusion

In conclusion, the present meta-analysis confirms that the patients before treatment with high SUV ^18^FDG PET/CT images and patients with metabolically nonresponding ^18^FDG PET/CT images may be considered at high risk of death or treatment failure. Therefore, the SUV of ^18^ FDG PET/CT is a useful tool to help predict survival outcomes in colorectal cancer patients with liver metastases.

## Additional files

Additional file 1: Figure S1. Funnel graph for the assessment of potential publication bias in studies about ΔSUV for EFS. (DOCX 17 kb) Additional file 2: Figure S2. Funnel graph for the assessment of potential publication bias in studies about ΔSUV for OS. (DOCX 17 kb) Additional file 3: Figure S3. Funnel graph for the assessment of potential publication bias in studies about pretreament SUV for OS. (DOCX 17 kb) Additional file 4: Figure S4. Funnel graph for the assessment of potential publication bias in studies about posttreament SUV for OS. (DOCX 23 kb)

## Abbreviations

^18^FDG PET/CT: 18-fluorodeoxyglucose positron emission tomography CT
^90^Y: Yttrium-90
ΔSUV: Follow-up SUV values
CI: Confidence intervals
DFS: Disease-free survival
EFS: Event-free survival
HR: Hazard ratio
OS: Overall survival
PFS: Progression-free survival
RFS: Relapse-free survival
SIRT: Selective internal radiation therapy
SUV: Standardized uptake value
