# Supplementary material for: Prognostic significance of 18FDG PET/CT in colorectal cancer patients with liver metastases: a meta-analysis
Source: Cancer Imaging. 2015 Nov 20;15:19. doi: 10.1186/s40644-015-0055-z (PMC4654916; doi:10.1186/s40644-015-0055-z)

Figure S4: Funnel graph for the assessment of potential publication bias in studies about posttreament SUV for OS.


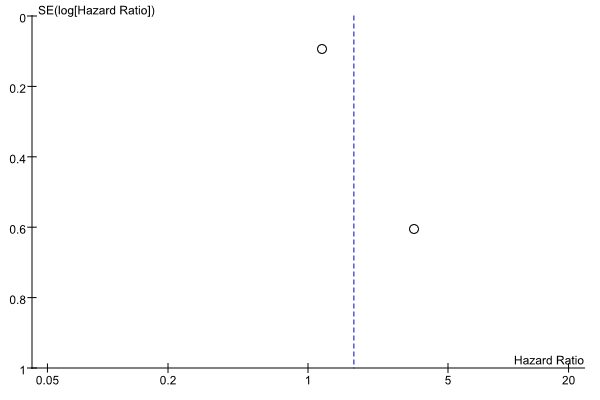

Supplement: Additional file 4: Figure S4. — Funnel graph for the assessment of potential publication bias in studies about posttreament SUV for OS. (DOCX 23 kb) [file 40644_2015_55_MOESM4_ESM.docx]
